# Using global isotopic data to constrain the role of shale gas production in recent increases in atmospheric methane

**DOI:** 10.1038/s41598-020-61035-w

**Published:** 2020-03-06

**Authors:** Alexei V. Milkov, Stefan Schwietzke, Grant Allen, Owen A. Sherwood, Giuseppe Etiope

**Affiliations:** 1 Department of Geology and Geological Engineering, Colorado School of Mines, Golden, CO USA; 2 Environmental Defense Fund, Berlin, Germany; 3 Centre for Atmospheric Science, The University of Manchester, Oxford Road, Manchester, M13 9PL UK; 4 Department of Earth and Environmental Sciences, Dalhousie University, Halifax, NS Canada; 5 Istituto Nazionale di Geofisica e Vulcanologia, Rome, Italy and Faculty of Environmental Science and Engineering, Babes-Bolyai University, Cluj-Napoca, Romania

**Keywords:** Solid Earth sciences, Biogeochemistry, Carbon cycle, Chemistry

## Abstract

The accelerated increase in global methane (CH_4_) in the atmosphere, accompanied by a decrease in its ^13^C/^12^C isotopic ratio (*δ*^13^C_CH4_) from −47.1‰ to −47.3‰ observed since 2008, has been attributed to increased emissions from wetlands and cattle, as well as from shale gas and shale oil developments. To date both explanations have relied on poorly constrained *δ*^13^C_CH4_ source signatures. We use a dataset of *δ*^13^C_CH4_ from >1600 produced shale gas samples from regions that account for >97% of global shale gas production to constrain the contribution of shale gas emissions to observed atmospheric increases in the global methane burden. We find that US shale gas extracted since 2008 has volume-weighted-average *δ*^13^C_CH4_ of −39.6‰. The average *δ*^13^C_CH4_ weighted by US basin-level measured emissions in 2015 was −41.8‰. Therefore, emission increases from shale gas would contribute to an opposite atmospheric *δ*^13^C_CH4_ signal in the observed decrease since 2008 (while noting that the global isotopic trend is the net of all dynamic source and sink processes). This observation strongly suggests that changing emissions of other (isotopically-lighter) CH_4_ source terms is dominating the increase in global CH_4_ emissions. Although production of shale gas has increased rapidly since 2008, and CH_4_ emissions associated with this increased production are expected to have increased overall in that timeframe, the simultaneously-observed increase in global atmospheric CH_4_ is not dominated by emissions from shale gas and shale oil developments.

## Introduction

Methane (CH_4_) is the third-most important greenhouse gas (after water vapor and carbon dioxide) and a significant contributor to global climate change^1–8^. Its globally averaged marine surface annual mean mole fraction in the atmosphere steadily increased from ~1600 parts per billion (ppb) to ~1775 ppb in the 1980–1990s, stabilized around ~1775 ppb during the period 1999–2006, and then returned to the earlier pattern of increases leading to ~1860 ppb in 2018^1,3,9,10^. There are anthropogenic (e.g., agriculture, wastes, fossil fuels, biomass burning) and natural (e.g., wetlands, freshwaters, geological seepage, wild fires) sources of CH_4_ to the atmosphere. The carbon-isotopic composition of CH_4_ (the ratio of stable isotopes ^12^C and ^13^C expressed as *δ*^13^C (‰) relative to the Vienna Pee Dee Belemnite standard), together with estimates of flux from each source-type, can be used to infer the relative contributions of various CH_4_ emitters to the global budget by matching co-constrained global observations of CH_4_ and its *δ*^13^C^3,4^. Furthermore, temporal variations in *δ*^13^C of atmospheric CH_4_ are a highly useful indicator of changes in the trends of various emitters and/or CH_4_ sinks. The global mean atmospheric *δ*^13^C_CH4_ trended upward between 1980 (approximately −47.7‰) and 1997 (approximately −47.1‰), and remained relatively stable until 2008, before decreasing towards the most recent values of approximately −47.3‰^1,6^.

This recent increase in the atmospheric CH_4_ burden, coincident with the depletion of ^13^C, has been attributed to the increasing contribution of biogenic CH_4_ from wetlands and from agricultural activities such as cattle husbandry that produce CH_4_ with *δ*^13^C usually more negative than −55‰^1,4,9^. While atmospheric CH_4_ sinks are less extensively studied, changes in the sink strength may at least partially explain some of the long-term observed trends in *δ*^13^C_CH4_^11^. Fossil fuel production also contributes to increasing atmospheric CH_4_^5,12,13^. However, CH_4_ in fossil fuels is, on average, enriched in ^13^C (*δ*^13^C = −44‰^4,14^) relative to globally-averaged atmospheric CH_4_. Decreasing *δ*^13^C of atmospheric CH_4_ since 2008 implies that emissions from biogenic sources are therefore increasing at a greater rate relative to emissions from fossil fuels. However, recent studies have suggested that emissions from conventional petroleum developments^5^ and from shale gas/oil developments in particular^2^ (see Text S1 in Supplementary Information) have been the greatest single cause of the recent global increase of atmospheric CH_4_. Here, we use a large global dataset of *δ*^13^C_CH4_ from produced shale formations, which leads us to conclude that emissions from shale gas and oil production have not played a dominant role in the increase in atmospheric CH_4_ since 2008.

## Materials and Methods

### Global isotopic dataset

We analyzed *δ*^13^C_CH4_ data for 1619 samples of produced natural gas from 38 shale formations around the world originally presented in 73 studies (Table S1). This shale gas dataset is a subset of a larger global inventory of gas samples from conventional reservoirs, shales, coals, seeps and other geological settings originally published by Sherwood *et al*.^14^ and further expanded and discussed by Milkov and Etiope^15^ and Milkov *et al*.^16^. Although most gas samples come from formations dominated by true shale lithology (e.g., the Marcellus Formation, USA), we also include samples collected from unconventional low-permeability (tight) reservoirs dominated by very fine-grained sandstone or siltstone (e.g., the Montney Formation, Canada) or mixed clastic/carbonate lithologies (e.g., the Niobrara Formation, USA) developed through hydraulic fracturing and commonly included in the inventories of produced shale gas^17^. The produced gas may be free gas associated with relatively little condensate liquids (e.g., in the Haynesville Formation) or oil-dissolved gas (e.g., in the Eagle Ford Formation). Most samples come from the USA (n = 1238), followed by China (n = 252), Canada (n = 124), United Kingdom (n = 2), Sweden (n = 2) and Australia (n = 1).

### Calculation of weighted δ^13^C_CH4_ values

Values of production volume-weighted *δ*^13^C_CH4_ for shale gases were derived by first calculating the proportion of gas production from each shale formation in the total production, then multiplying that value by the average *δ*^13^C_CH4_ for the corresponding shale formation, and then summing up the results. Emission volume-weighted *δ*^13^C-CH_4_ values were derived by first calculating the proportion of CH_4_ emissions from each shale formation in total emissions, multiplying that value by average *δ*^13^C_CH4_ for corresponding shale formation, and then summing up the results.

## Results

**Figure 1:**
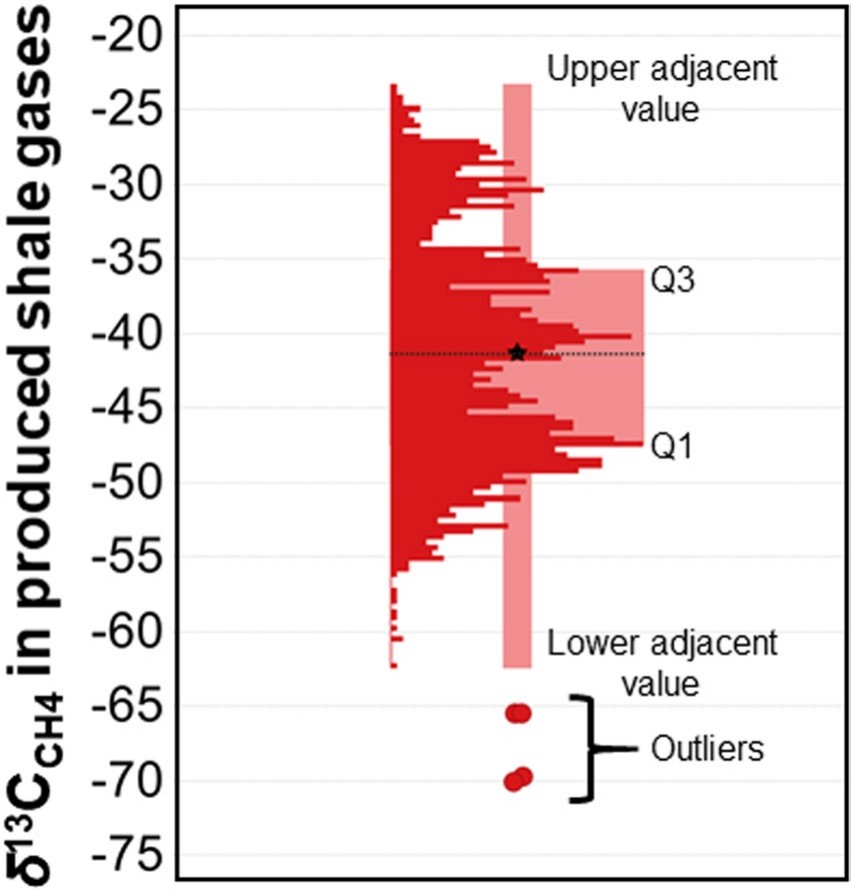
δ^13^C_CH4_ values from a global dataset of 1619 samples of produced shale gases from around the world. The data are displayed using a box plot, which shows distribution of values as histogram, average (mean) value (−41.3‰) as black star, median value (−41.4‰) as dotted line, first quartile (Q1), third quartile (Q3), lower adjacent value, upper adjacent value, and outliers. The first quartile (Q1) is the median of the lower half of the data set. This means that about 25% of the values in the data set lie below Q1 and about 75% lie above Q1. The third quartile (Q3) is the median of the upper half of the data set. This means that about 75% of the values in the data set lie below Q3 and about 25% lie above Q3. The lower adjacent value is the smallest observation that is greater than or equal to the lower inner fence, which is the first quartile minus 1.5 × IQR, where IQR stands for the interquartile range. The upper adjacent value is the largest observation that is less than or equal to the upper inner fence, which is the third quartile plus 1.5 × IQR. Outliers are all values that fall outside of either of the fences. Original data are in Table S1.

The arithmetic mean *δ*^13^C_CH4_ for all shale gas samples is −41.3 ± 0.2‰ (n = 1619, range from −70‰ to −23.3‰, median −41.4‰) (Fig. 1). The mean value is slightly more positive than −42.5 ± 0.3‰ reported by Sherwood *et al*.^14^ based on a smaller dataset of 647 samples. Methane from produced shales is, on average, more enriched in ^13^C than CH_4_ produced from conventional oil and gas reservoirs (mean *δ*^13^C_CH4_ = −44.0 ± 0.1‰, n = 6079 in the study of Sherwood *et al*.^14^; mean *δ*^13^C_CH4_ = −42.8 ± 0.1‰, n = 12,697 in the study of Milkov *et al*.^16^) and significantly more enriched in ^13^C than the modern atmospheric *δ*^13^C_CH4_ (−47.3‰^1,6^). We note that shale gas is even more enriched in ^13^C relative to the global average *δ*^13^C_CH4_ (about −54‰^4^) of all atmospheric sources prior to isotopic fractionation of atmospheric CH_4_ by all sinks resulting in the modern atmospheric value above.

**Figure 2:**
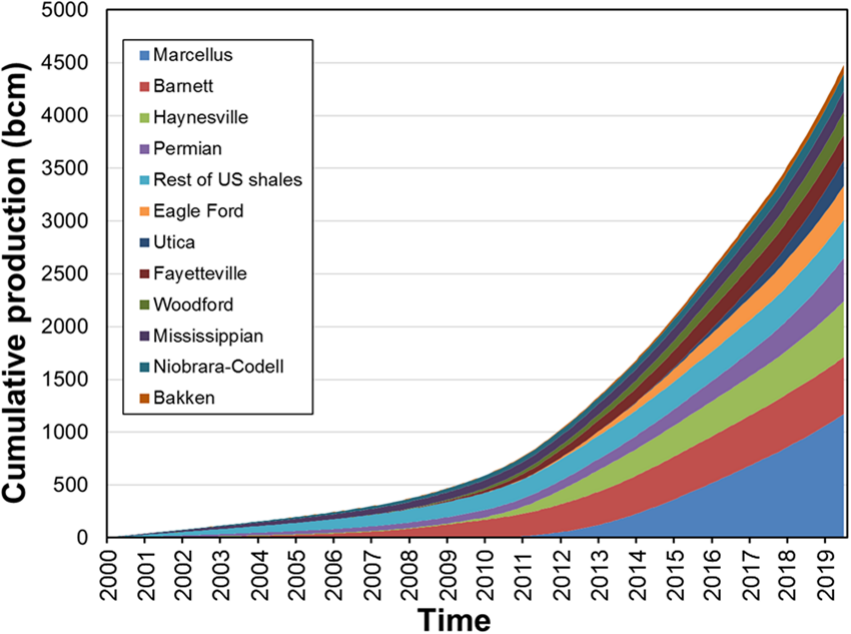
Cumulative production (in billion cubic meters or bcm) of dry gas from shale plays in the USA. Data are from Energy Information Administration^17^.

**Figure 3:**
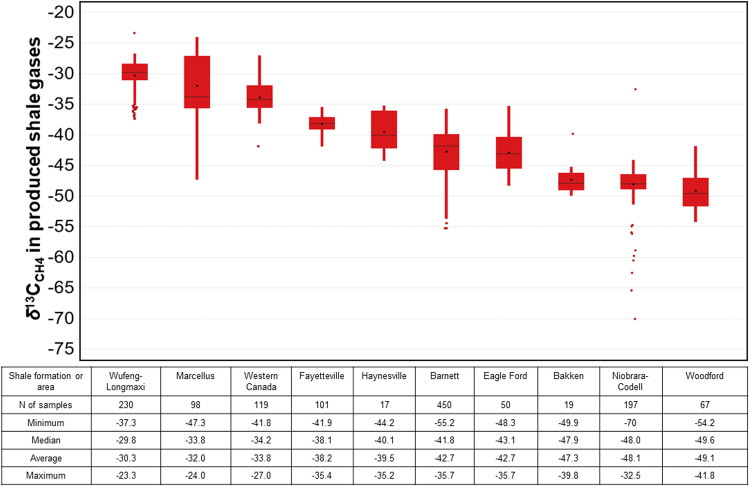
Main statistics on *δ*^13^C_CH4_ for gases produced from main shale formations in the USA, Canada and China. The data are displayed using box plots (see Fig. 1 for legend). Original data are in Table S1.

Global shale gas production increased from about 31 billion cubic meters (bcm) in 2005 to about 434 bcm in 2015^18^. In the USA, the cumulative production of shale gas from 2000 to mid-2019 reached approximately 4.5 trillion cubic meters (tcm), including about 4.1 tcm produced since 2008 (Fig. 2, based on dry gas production). Half of the cumulative shale gas was produced from the Marcellus, Barnett and Haynesville formations. Figure 3 summarizes *δ*^13^C_CH4_ data on gases produced from these and other principal shale formations in the USA.

In this study, we use the global *δ*^13^C_CH4_ dataset to derive *δ*^13^C_CH4_ representative of both produced gas (volume-weighted average) and the *δ*^13^C_CH4_ signature when weighted for measured emissions across plays (emission-weighted average). Table 1 presents average *δ*^13^C_CH4_ for the main producing shale plays in the USA. The 1002 available gas samples with *δ*^13^C_CH4_ data are from plays that account for 94% of cumulative US shale gas production. The average *δ*^13^C_CH4_, when weighted by the amount of cumulative production from each shale play during 2008–2019, is −39.6‰. A large proportion (28%) of cumulative shale gas production comes from the Marcellus Formation where CH_4_ is significantly enriched in ^13^C (mean *δ*^13^C_CH4_ is −32.0‰, n = 98). This latter source significantly influences the average volume-weighted isotope signature of CH_4_ produced from shales in the USA.

**Table 1:** Data used to calculate the average *δ*^13^C_CH4_ in shale gases produced in the USA since 2008.

Shale formation	Total dry shale gas production from 2008 to mid-2019 (bcm)	Portion (%) of total dry shale gas production from 2008 to mid-2019	Average *δ*^13^C_CH4_ in produced gas (‰)	N of gas samples with *δ*^13^C_CH4_
Marcellus	1170	28	−32.0	98
Haynesville	520	13	−39.5	17
Barnett	460	11	−42.7	450
Permian basin (Wolfcamp, Avalon and others)	360	9	−49.5	13
Rest of US shales	236	6	na	na
Eagle Ford	319	8	−42.9	50
Utica	245	6	−31.8	4
Fayetteville	240	6	−38.2	101
Woodford (Anadarko and Arkoma basins)	210	5	−49.1	54
Mississippian (Anadarko basin)	137	3	−50.3	7
Niobrara-Codell (Denver basin)	133	3	−47.6	190
Bakken	85	2	−47.3	19
**Total**	**4114**	**100**		**1003**
**Total with gas data**	**3879**	**94**		
**Production volume-weighted**			**−39.6**	

Production data are from Energy Information Administration^17^. See Text S2 in Supplementary Information for specific details on Permian, Utica and Niobrara-Codell samples. na – not available.

**Figure 4:**
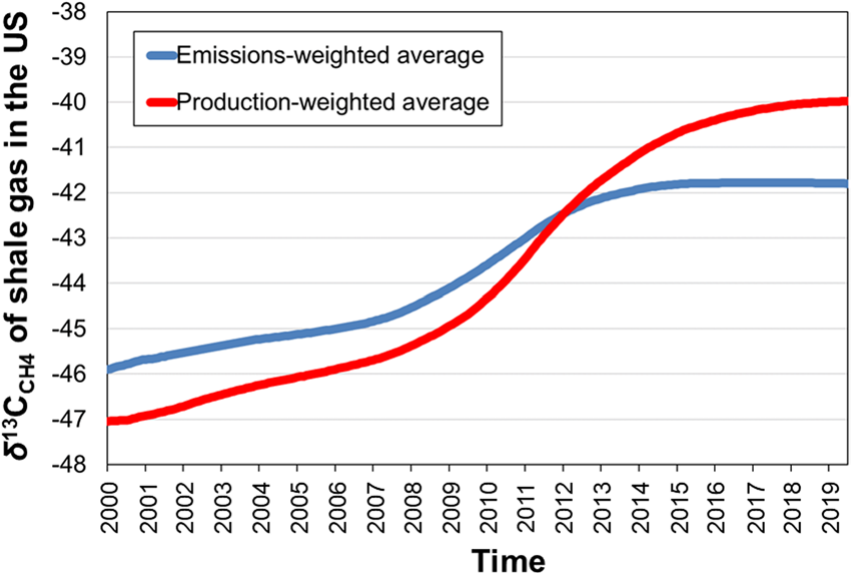
Increasing *δ*^13^C_CH4_ signature of produced and emitted gas from shale developments in the USA from 2000 to mid-2019. Only formations with sufficient isotopic data (Marcellus, Barnett, Haynesville, Permian, Eagle Ford, Fayetteville, Woodford, Niobrara-Codell and Bakken) and emission data (Marcellus, Barnett, Haynesville, Eagle Ford, Fayetteville, Niobrara-Codell and Bakken) are used to construct this figure.

The average shale *δ*^13^C_CH4_ weighted by the amount of emissions measured in 2015 from the main USA shale plays^19^ is −41.8‰ (Table 2). Sensitivity analysis suggests that this value changes little when emission measurements from other years are considered (see Table S2, Text S3). We also calculated how the average *δ*^13^C_CH4_ signature of shale-emitted gas changed over time. When weighted by production or emissions, the US average signature becomes heavier (thus, opposite to the direction of the atmospheric trend) by about 4–7‰ from 2000 to mid-2019 (Fig. 4). This is because the relative contribution of shales with relatively more positive *δ*^13^C_CH4_ (e.g., Marcellus and Haynesville formations) to both production and emissions increased in that period.

**Table 2:** Data used to calculate the emission-weighted average *δ*^13^C of CH_4_ emitted from, mostly, shale gas production in selected plays and areas in the USA in 2015.

Shale formation and basin/area	Gas production (m^3^/d)	Emitted gas (% of production)	CH_4_ emissions (tones/hr)	Average δ^13^C (‰) of emitted CH_4_
Eagle Ford	1.54E + 08	2.5	83	−42.9
Haynesville	1.50E + 08	1	42	−39.5
Marcellus (N.E. PA)	1.40E + 08	0.4	18	−32.0
Barnett	1.20E + 08	1.5	46	−42.7
Fayetteville	6.80E + 07	1.5	31	−38.2
Niobrara-Codell (Denver)	3.80E + 07	2.1	18	−47.6
Bakken	3.70E + 07	5.4	29	−47.3
**Total**	**7.07E** **+** **08**		**267**	
**Volume-weighted average**		**1.6**		
**Emission-weighted average**				**−41.8**

Gas production, percentage of emitted gas, and CH_4_ emissions are from Peischl *et al*.^19^ and references therein. These results account for ~60% of total USA shale gas production in 2015.

Gas samples from three other countries currently producing shale gas commercially (Canada, China, Argentina) indicate somewhat more positive *δ*^13^C_CH4_ values than the USA (Fig. 3), resulting in a global volume-weighted *δ*^13^C_CH4_ signature of −38.8‰ (Table S3, Text S4). The volume- and emission-weighted *δ*^13^C_CH4_ values calculated here do not account for shale plays for which production has become negligible after the 1990s (see Text S4).

The US shale *δ*^13^C_CH4_ weighted by the amount of cumulative production from 2000 to mid-2019 for each shale play is −40.0‰ (Table S4). The mean *δ*^13^C_CH4_ of produced shale gas in the USA since 2008 is −39.6‰ (Table 1). The slight (by 0.4‰) enrichment in ^13^C during 2008–2019, relative to 2000–2019, is due to a relatively larger contribution of production from the Marcellus and Haynesville formations in 2008–2019, and a smaller contribution from the Barnett Formation during that period. The mean *δ*^13^C_CH4_ of globally produced shale gas in 2018 is −38.‰ (Table S3). We also estimated an emission-weighted *δ*^13^C_CH4_ of −41.8‰ for the principal US shale plays in 2015 (Table 2). These values are appropriate for utilization in models that constrain CH_4_ emissions from shale developments to the atmosphere based on matching modelled global *δ*^13^C to observed *δ*^13^C. Methane from produced shales is, on average, significantly enriched in ^13^C relative to atmospheric CH_4_ (*δ*^13^C_CH4_ ~−47‰).

## Discussion

Recently, atmospheric CH_4_ became more abundant but also depleted in ^13^C, as *δ*^13^C decreased from about −47.1‰ in 2007 to −47.3‰ in 2017. If shale gas (with *δ*^13^C_CH4_ around −40‰ as documented in this study) and conventional oil and gas (with *δ*^13^C_CH4_ around −43‰^16^) were conceived to collectively dominate recent emissions of CH_4_ to the atmosphere, then atmospheric CH_4_ would very simply become more enriched in ^13^C relative to the current global mean *δ*^13^C, which is not consistent with global observations. While we agree that shale developments (and fossil fuel in general) represent an important CH_4_ source, and that emissions from those sources have been likely increasing due to growing production, we conclude that the increases in global atmospheric CH_4_ concentrations since 2008 are not as strongly attributable to shale gas and conventional oil and gas emissions as some studies claim^2,5^, based on our global observations of isotopic fractionation.

Additionally, we must emphasize that the measured atmospheric *δ*^13^C_CH4_ signal is the sum-total of all CH_4_ source and sink terms. For example, a decrease in biomass burning emissions (significantly enriched in ^13^C (*δ*^13^C_CH4_ −22.3 ± 1.9‰^4^), and an increase in fossil fuel emissions (including shale gas), could in principle result in the same global average atmospheric *δ*^13^C_CH4_ signal over time as if both sources had no trend^4,5^. The biomass burning category includes fires and solid biofuels (e.g., for use in cook stoves). Data on global CH_4_ emissions from fires is not entirely conclusive. Remote sensing data of CH_4_ and CO (and assuming (i) biomass burning CH_4_/CO emission ratios and (ii) a partitioning of CO emissions across sectors) suggests decreased fire CH_4_ emissions of ~3.7 Tg/yr from the 2001–2007 to the 2008–2014 periods^5^. In contrast, remote sensing of burned fire area suggests no such trend^20^ (no trend over this period apart from inter-annual variation; Fig. S1). Furthermore, CH_4_ emissions from solid biofuels are reported to have increased from 12.2 to 13.6 Tg/yr from 2000–2012^21^ (latest time series available). While this data does not indicate an immediately apparent decrease in global biomass burning CH_4_ emissions, more research is needed. Potential trends in the various CH_4_ sink processes such as the soil sink^22^ and the tropospheric OH sink^11^ can further complicate the diagnosis of source trends. As a result, it is important to account for these processes, as well as other existing evidence such as latitudinal and seasonal CH_4_ trends, when attributing the global signal^1,9^.

From the above, it follows that attributing ~1/3 of the global CH_4_ increase to North American shale gas production and another ~1/3 to conventional gas and oil with a simple mass balance approach^2^ is not supported by observations because of unconstrained uncertainties. Based on long-term airborne CH_4_ measurements over the US, previous analysis concludes that oil and gas industry CH_4_ emissions (shale and conventional) over the past decade have increased at about the same rate as natural gas production volume^7^. The existence of unaccounted and poorly characterized emission sources within the oil and gas industry has also been demonstrated through intensive field studies in the USA^23^, and additional international studies paint a similar picture^24,25^, although little independent measurement data exist for many world regions including the Middle East, the Former Soviet Union, and Africa. Further research targeted for these areas, in addition to changing biogenic sources and sinks, will serve to further constrain the conclusions made in this work.

Based on existing knowledge of CH_4_ source and sink terms and isotopic signatures, additional CH_4_ emissions associated with increased shale gas development in the USA cannot account for a large fraction of the recent increase in atmospheric CH_4_. Yet, oil and gas industry expansion remains a significant factor in the complex patterns of global atmospheric CH_4_ emissions and concentrations^4,23–25^. And, of equal importance, fossil fuel CH_4_ sources may be mitigated with policy and best (or better) industrial practice that can effectively reduce emissions. We suggest that the rise in global CH_4_ concentrations is most effectively seen not through a lens of what is the most important or dominant source of emissions, but rather understanding all sources and how they can collectively explain the observed patterns of atmospheric increases. Indeed, a reduction in emissions from any major source (such as fossil fuels or cattle husbandry) would be expected to lead to a reduction in the global CH_4_ concentration^1^. Therefore, although our analysis indicates that shale gas and conventional gas and oil production has not played a dominant role in the increase in atmospheric CH_4_ since 2008, we should not lose sight of the powerful impact of interventions to reduce emissions from sources we have.

## Conclusions

CH_4_ recently increased in the atmosphere and simultaneously became more depleted in ^13^C. In this study, we compiled a large global dataset of isotopic composition of CH_4_ produced from shale formations that account for most global shale gas production. Developments of shale gas and oil on average emit CH_4_ significantly more enriched in ^13^C than the atmospheric CH_4_ signal. Given current knowledge of global isotopic data and processes, the increase in US shale oil and gas apparently does not dominate the recent increased emissions of global CH_4_ to the atmosphere. It is important to understand all sources of CH_4_ that collectively contribute to recent atmospheric increases, and isotopic data provide key constraints for this.

## Data Availability

The dataset used in this study is available as Supplementary information.

## Supporting Information

Supporting Information.
Supplementary Dataset.
