# Supplementary material for: Using global isotopic data to constrain the role of shale gas production in recent increases in atmospheric methane
Source: Sci Rep. 2020 Mar 6;10:4199. doi: 10.1038/s41598-020-61035-w (PMC7060170; doi:10.1038/s41598-020-61035-w)
Supplement: Supplementary file 1 — Supporting Information. [file 41598_2020_61035_MOESM1_ESM.docx]

**Using global isotopic data to constrain the role of shale gas production in recent increases in atmospheric methane**

Alexei V. Milkov^1^, Stefan Schwietzke^2^, Grant Allen^3^, Owen A. Sherwood^4^, Giuseppe Etiope^5^

^1^Department of Geology and Geological Engineering, Colorado School of Mines, Golden, CO, USA

^2^Environmental Defense Fund, Berlin, Germany

^3^Centre for Atmospheric Science, The University of Manchester, Oxford Road, Manchester M13 9PL, UK

^4^Department of Earth and Environmental Sciences, Dalhousie University, Halifax, NS, Canada

^5^Istituto Nazionale di Geofisica e Vulcanologia, Rome, Italy and Faculty of Environmental Science and Engineering, Babes-Bolyai University, Cluj-Napoca, Romania

Corresponding author: Alexei V. Milkov ([amilkov@mines.edu](mailto:amilkov@mines.edu))

**Content of this file**

Tables S1 to S4

Figure S1

Texts S1 to S4

References

**Table S1.** Samples of produced shale gases with *δ*^13^C_CH4_ used in this study. This is a truncated example. See Excel file (part of Supplementary Information) for the entire dataset.


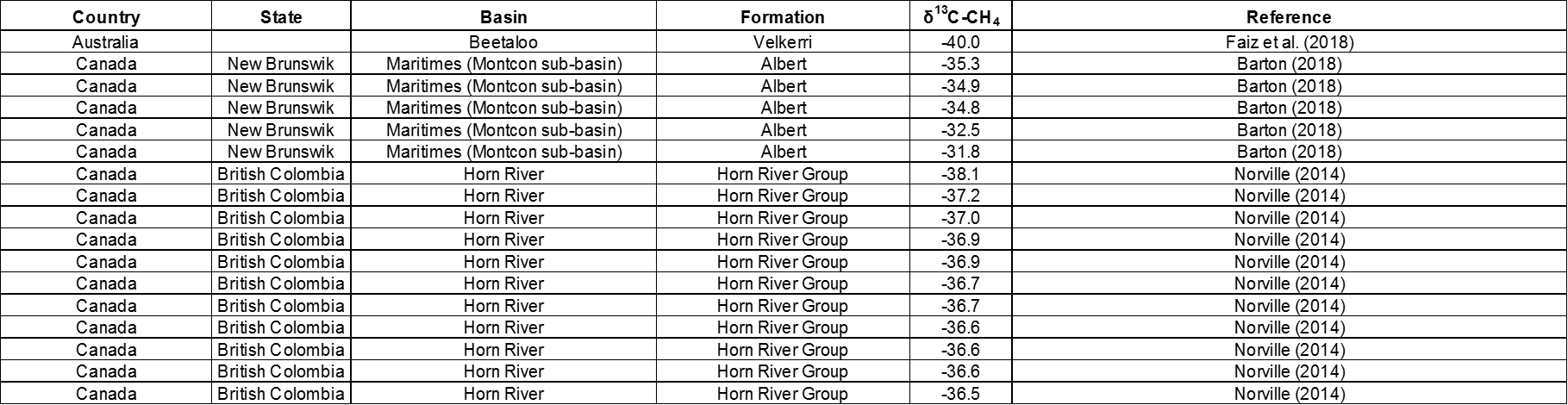


**Table S2.** Data used to calculate the emission-weighted average *δ*^13^C of CH_4_ emitted from, mostly, shale gas production in selected plays and areas in the USA in 2012-2015. Gas production and percentage of emitted gas are from Peischl et al. (2018), Peischl et al. (2016), Peischl et al. (2015), Schwietzke et al. (2017), Karion et al. (2015), Barkley et al. (2017), Pétron et al. (2014).

**Table S3.** Data used to calculate the mean *δ*^13^C_CH4_ value for recently (2018) produced shale gas in the world. Production data are from EIA, 2019 (USA), National Energy Board, 2018 (Canada), S&P Global, 2019 (China) and EIA, 2019 (Argentina). na – not available.

**Table S4.**  Data used to calculate the average *δ*^13^C_CH4_ in shale gases produced in the USA from 2000 to mid-2019. Production data are from Energy Information Administration (2019). na – not available.

**Figure S1.** Global CH_4_ emissions from all fire types combined. Data are from the Global Fire Emissions Database (<https://www.geo.vu.nl/~gwerf/GFED/GFED4/tables/GFED4.1s_CH4.txt>).

**
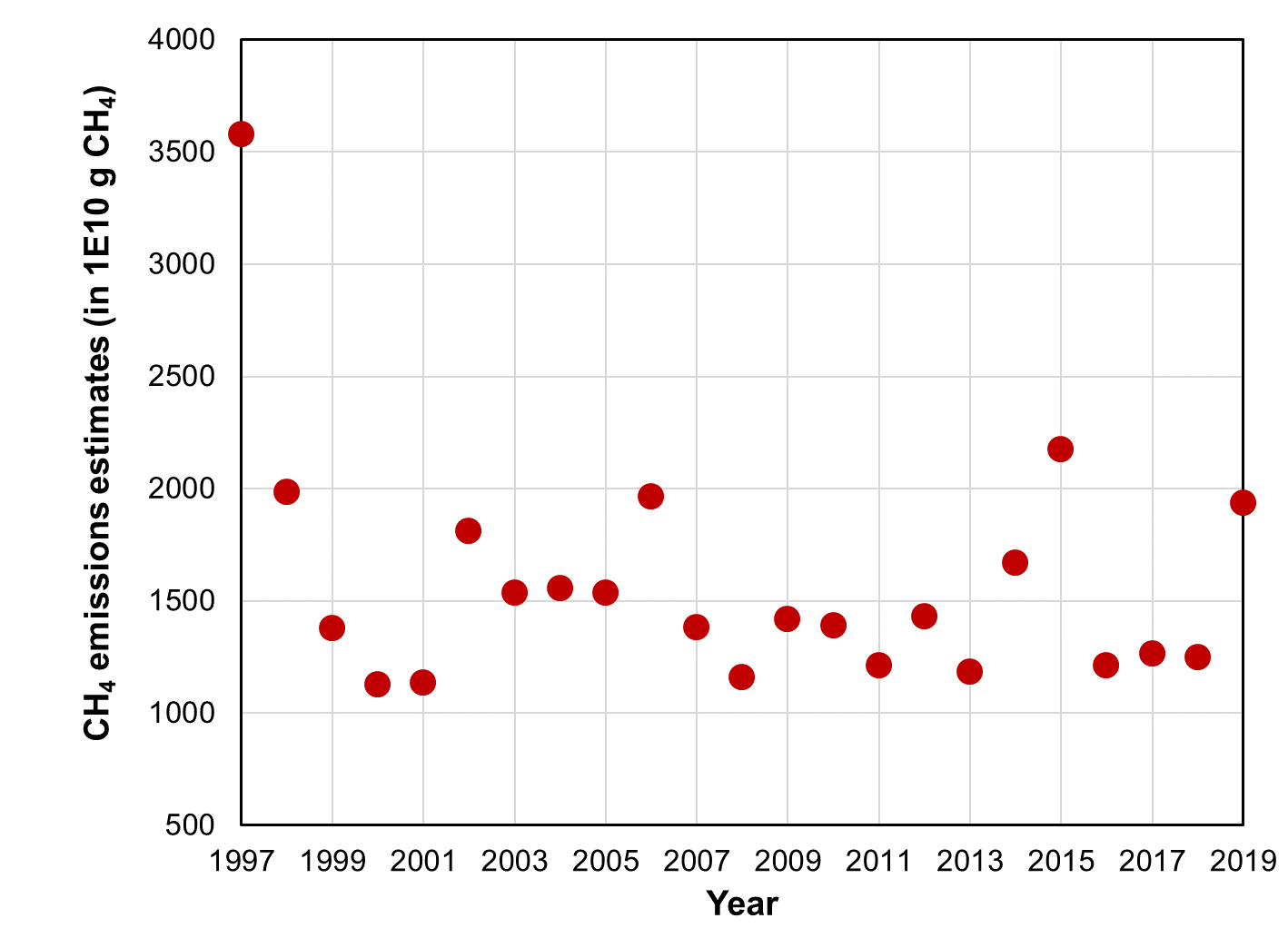
**

**Text S1. Non-representativeness of shale gas data in the study of Howarth (2019)**

Howarth (2019) estimated average *δ*^13^C_CH4_ -46.9‰ for produced shale gases. This value was based on data for 43 gas samples presented in three published studies. Nine samples were from producing wells in the Utica Formation (one sample, Botner et al., 2018) and in the Bakken Formation (eight samples, Schoell et al., 2011). The other 34 samples were air samples taken at the surface near natural gas well pads in the area of the Barnett Formation (Townsend-Small et al., 2015). Below we explain why the average *δ*^13^C_CH4_ -46.9‰ is not reprehensive of US- and globally-produced shale gases.

The sample from the Utica formation with *δ*^13^C_CH4_ -47.3‰ taken in Ohio was not representative of the total shale gas produced from that formation. A significant portion of gas from the Utica Formation is produced in Pennsylvania, where shale is thermally mature, gas is dry (enriched in CH_4_) and has *δ*^13^C_CH4_ often more positive than -30‰ (e.g., -25.7‰ reported in Wang et al. (2015) and -27.0‰ reported in Burruss and Laughrey (2010)). In general, *δ*^13^C_CH4_ in shale plays depend on thermal maturity of shales (Milkov and Etiope, 2019), which may vary widely across the play area as a function of maximum burial depth and resulting thermal stress (maturity) of the formation. This highlights the need to weight *δ*^13^C_CH4_ for production volumes and emission volumes as we did in this study.

In addition to the small sample size, the Utica, Bakken and Barnett formations account for only ~15% of global shale gas production in 2018 (Energy Information Administration, 2019). There are many shales that have *δ*^13^C_CH4_ more positive than produced gas from those formation. For example, the Marcellus Formation is the major producer, which contributed 27% to total global shale gas production in 2018, and it has average *δ*^13^C_CH4_ of -31.9‰ (n=97). Average *δ*^13^C-CH_4_ values weighted for the volumes of produced gas and emitted gas are more appropriate to use in the models of emissions from shales than simple average values.

**Text S2. Specific details on average *δ*^13^C_CH4_ (Tables 1 and 2) for gas in some shale plays**

For the Permian basin, only mud gas data collected from the Avalon and Wolfcamp formations during drilling (IsoTubes) are available (Goldsmith and Abrams, 2016). Although these are not production gas samples, gases collected into IsoTubes during drilling have essentially the same *δ*^13^C_CH4_ as produced gases from the same formation, although this has been demonstrated only in studies of conventional reservoirs (e.g., Ellis et al., 2007; Milkov et al., 2007; Petersen et al., 2019).

There are only four gas samples available in the Utica Formation with *δ*^13^C_CH4_ -25.7‰ (Wang et al., 2015), -27.0‰ (Burruss and Laughrey, 2010), -27.3‰ (Pennsylvania Geological Survey, 2007; IsoTube sample taken during drilling), and -47.3‰ (Botner et al., 2018). This range reflects varying thermal maturity of the Utica Formation across the play area. The Utica Formation is more mature and produces mostly dry gas (with more positive *δ*^13^C_CH4_) in Pennsylvania, and is less mature and produces oil and oil-associated gas in Ohio (with more negative *δ*^13^C_CH4_) (Patchen and Carter, 2015).

For the Niobrara-Codell play, only data from the Weld County (Colorado), where the vast majority of production and emission from this formation occurs, are included in Tables 1 and 2. Data from Rice and Claypool (1981) suggest that Niobrara gases have more negative *δ*^13^C_CH4_ (average -61.7‰, n=7, included in Figures 1 and 3) than in the Weld County, but these samples come from north-eastern Colorado and Kansas where the current shale gas production is negligible.

No gas data are reported for the “Rest of US shales” category because the shale formations are not specified.

**Text S3. Sensitivity analysis for emission-weighted average *δ*^13^C_CH4_ in the US**

The emission-weighted average *δ*^13^C of CH_4_ from shale plays in the US presented in Table 2 is based on gas fluxes measured in 2015 from aircraft (Peischl et al., 2018). However, aircraft-measured emission estimates have large daily variations, including uncertainties that decrease after multi-day measurements (see, for example, Karion et al., 2015). In Table S2, emission rates from multiple estimates for some basins in 2012-2015 are combined with reported production in 2015 to estimate absolute emissions, which are then used to weight isotopic signatures of shale-emitted CH_4_ within the US. When two emission rate estimates from two different years were available, we averaged the rates. This approach changes the emission-weighted average *δ*^13^C_CH4_ to -42.7‰ (because of greater assumed emissions from the Bakken and Niobrara-Codell plays). In addition, we used a Monte Carlo analysis to calculate the 1SD (one standard deviation) uncertainty around this average value, which is small (0.36‰).

**Text S4. Global signature of produced shale gas *δ*^13^C_CH4_**

Only four countries currently have commercial shale gas production. The USA is the dominant producer of shale gas in the world (about 1.87 bcm/day at the end-2018, EIA, 2019; accounting for ~85% of global shale and tight gas production). In Canada, unconventional shale and tight gas (about 0.28 bcm/day in 2018, National Energy Board, 2018) is produced in Alberta and British Columbia and has mean *δ*^13^C_CH4_ of -37.1‰ (range from –41.8‰ to -27.0‰, n=119). In China, shale gas (about 0.028 bcm/day in 2018, S&P Global, 2019) is produced mostly from the Wufeng-Longmaxi Formation in the Sichuan basin, with a mean *δ*^13^C_CH4_ of -30.3‰ (range from -37.3‰ to -23.3‰, n=230). In Argentina, shale gas (about 0.028 bcm/day at the end-2018, EIA, 2019) is produced from Jurassic shales (mostly the Vaca Muerta Formation) in the Neuquen basin with a mean *δ*^13^C_CH4_ of -41.3‰ (range from -57.1‰ to -31.1‰, n=39, limited to mud gas drilling data from five wells; Ostera et al., 2016; Porras et al., 2018). The mean *δ*^13^C_CH4_ from 1468 gas samples weighted by the amount of recent (year end-2018) global production from shale plays around the world is -38.8‰ (Table S3). These samples are from fields that account for 97% of global production.

There are shale plays not included in Tables 1-2 and S2-S4 which produce CH_4_ more depleted, on average, in ^13^C than the average values reported in those tables. For example, gas produced from the Antrim Formation in the Michigan basin (USA) has average *δ*^13^C_CH4_ of -51.3‰ (range from -57.9‰ to -43‰, n=77, Table S1) and gas produced from the New Albany Formation in the Illinois basin (USA) has average *δ*^13^C_CH4_ of -52.3‰ (range from -69.7‰ to -45‰, n=75, Table S1). Methane in these plays has early mature thermogenic and secondary microbial origin, and it was generated and preserved in smaller volumes than thermogenic gas in thermally mature and overmature plays such as the Marcellus Formation (Milkov and Etiope, 2019). The average *δ*^13^C_CH4_ for all shale gases in our dataset (-41.3‰) accounts for these shale formations with CH_4_ relatively depleted in ^13^C. However, contributions from these shale formations do not significantly affect the mean production volume-weighted and emission-weighted *δ*^13^C_CH4_ estimated in this study for the USA and the world. These naturally fractured shales were relatively important producers in the 1990s (Curtis, 2002), but recent annual production from them is negligible relatively to other shale plays (EIA, 2019).

**References**

Barkley, Z.R. *et al.* (2017) Quantifying methane emissions from natural gas production in North-Eastern Pennsylvania. *Atmos. Chem. Phys.* 17, 22, <https://doi:10.5194/acp-17-13941-2017>

Botner, E.C. et al. Monitoring concentration and isotopic composition of methane in groundwater in the Utica Shale hydraulic fracturing region of Ohio. *Environ. Monit. Assess.* 190, 322–337, <https://doi.org/10.1007/s10661-018-6696-1> (2018).

Burruss, R.C. & Laughrey, C.D. (2010) Carbon and hydrogen isotopic reversals in deep basin gas: Evidence for limits to the stability of hydrocarbons. *Org Geochem* 41, 1285-1296.

Curtis, J.B. (2012) Fractured shale-gas systems. *AAPG Bull* 86, 1921–1938.

Energy Information Administration (2019) Growth in Argentina’s Vaca Muerta shale and tight gas production leads to LNG exports. <https://www.eia.gov/todayinenergy/detail.php?id=40093> (Accessed on 22^nd^ August, 2019).

Ellis, L., Berkman, T., Uchytil, S. & Dzou, L. (2007) Integration of mud gas isotope logging (MGIL) with field appraisal at Horn Mountain Field, deepwater Gulf of Mexico. *J Petr Sci Engineer* 58, 443–463.

Goldsmith, M. & Abrams, M.A. (2016) Gas isotope analysis: A cost effective method to improve understanding of vertical drainage in the Delaware Basin. *Unc Res Techn Conf (URTeC)* DOI 2458154.

Karion, A. et al. (2015) Aircraft-based estimate of total methane emissions from the Barnett Shale region. *Environ Sci Technol* 49, 8124–8131.

Milkov, A.V. & Etiope, G. (2019) Origin of shale gases from around the world: Implications for exploration. *Unc Res Technol Conf* URTeC:465, DOI 10.15530/urtec-2019-465.

Milkov, A.V. et al. (2007) Compartmentalization and time-lapse geochemical reservoir surveillance of the Horn Mountain oil field, deep-water Gulf of Mexico. *AAPG Bulletin* 91, 847-876.

National Energy Board (2018) Energy Futures Supplement. Canada’s Energy Future 2018 Supplement: Natural Gas Production. NE2-16E-PDF, 35 p. Available at: <http://www.neb-one.gc.ca/nrg/ntgrtd/ftr/2018ntrlgs/index-eng.html> (Accessed on 22nd August, 2019).

Ostera, H.A., García, R., Malizia, D., Kokot, P., Wainstein, L. & Ricciutti, M. (2016) Shale gas plays, Neuquén Basin, Argentina: chemostratigraphy and mud gas carbon isotopes insights. *Braz J Geol* 46, 181-196.

Patchen, D.G. & Carter, K.M. (2015) A geological play book for Utica shale Appalachian basin exploration. Final report of Utica Shale Appalachian Basin Exploration Consortium, 205 p. Available at <http://www.wvgs.wvnet.edu/utica/playbook/docs/FINAL_UTICA_REPORT_07012015.pdf> (Accessed on 3rd October, 2019).

Petersen, H.I., Hillock, P., Milner, S., Pendlebury, M. & Scarlett, D. (2019) Monitoring gas distribution and origin in the Culzean field, UK Central North Sea, using data from a continuous isotope logging tool and IsoTube and test samples. *J Petr Geol* 42, 435-450.

Peischl, J. et al. (2016) Quantifying atmospheric methane emissions from oil and natural gas production in the Bakken Shale region of North Dakota. *J Geophys Res Atmos* 121, 6101–6111, <https://doi:10.1002/2015JD024631>

Peischl, J., et al. (2015) Quantifying atmospheric methane emissions from the Haynesville, Fayetteville, and Northeastern Marcellus Shale gas production regions. *J Geophys Res Atmos* 120, 2119–2139.

Porras, J.S., Rodríguez, R. & Naides, C. (2018) Isotopic and compositional inversions in gases from Vaca Muerta Formation: Implications in fluids, overpressure and production predictions. 10º Congreso de Exploración y Desarrollo de Hidrocarburos Simposio de Recursos No Convencionales: Hacia una Nueva Convención, Instituto Argentino del Petróleo y el Gas, pp. 23-41.

Rice, D.D. & Claypool, G.E. (1981) Generation, accumulation, and resource potential of biogenic gas. *AAPG Bulletin* 61, 5-25.

Schoell, M., Lefever, J. A. & Dow, W. Use of maturity-related changes in gas isotopes in production and exploration of Bakken shale plays. AAPG Search and Discovery Article 90122, <http://www.searchanddiscovery.com/abstracts/pdf/2011/hedberg-beijing/abstracts/ndx_schoell.pdf> (2011).

S&P Global (2019) Analysis: China to miss 2020 shale gas production targets amid tough upstream conditions. Available at: <https://www.spglobal.com/platts/en/market-insights/latest-news/oil/043019-analysis-china-to-miss-2020-shale-gas-production-targets-amid-tough-upstream-conditions> (Accessed on 22nd August, 2019).

Townsend-Small, A. et al. Integrating source apportionment tracers into a bottom-up inventory of methane emissions in the Barnett shale hydraulic fracturing region. *Environ. Sci. Technol.* 49, 8175–8182, <https://doi.org/10.1021/acs.est.5b00057> (2015).
